# Justifying the More Restrictive Alternative: Ethical Justifications for One Health AMR Policies Rely on Empirical Evidence

**DOI:** 10.1093/phe/phac025

**Published:** 2022-11-07

**Authors:** Tess Johnson, William Matlock

**Affiliations:** Oxford Uehiro Centre for Practical Ethics, University of Oxford, Oxford, UK; Ethox Centre, University of Oxford, Oxford, UK; Nuffield Department of Medicine, University of Oxford, Oxford, UK

## Abstract

Global consumption of antibiotics has accelerated the evolution of bacterial antimicrobial resistance. Yet, the risks from increasing bacterial antimicrobial resistance are not restricted to human populations: transmission of antimicrobial resistant bacteria occurs between humans, farms, the environment and other reservoirs. Policies that take a ‘One Health’ approach deal with this cross-reservoir spread, but are often more restrictive concerning human actions than policies that focus on a single reservoir. As such, the burden of justification lies with these more restrictive policies. We argue that an ethical justification for preferring One Health policies over less restrictive alternatives relies on empirical evidence as well as theory. The ethical justification for these policies is based on two arguments: (i) comparatively greater effectiveness, and (ii) comparatively better tracking of moral responsibility. Yet the empirical assumptions on which these claims rest are limited by existing empirical knowledge. Using livestock farming as an example, we suggest that scientific research into characterising antimicrobial resistance and linking practices to outcomes ought to be guided (at least in part) by the imperative to supply the context-specific data needed to ethically justify preferring a One Health policy over less restrictive alternatives.

## Introduction

Global consumption of antibiotics has accelerated the evolution of bacterial antimicrobial resistance (AMR).^1^ AMR can be found in both anthropogenic (e.g. nosocomial, community, wastewater, agricultural) and non-anthropogenic (e.g. waterways, oceans, soil) reservoirs in which bacteria can live. Between these reservoirs, antimicrobial-resistant bacteria can spread, as well as AMR genes and antimicrobials themselves (Wright, 2007). This allows AMR that arises in bacteria in one reservoir (e.g. due to antimicrobial use there) to disseminate into other reservoirs through movement of the bacteria, horizontal transmission of the genes, or use of the antimicrobials themselves, causing resistance across reservoirs. Given the threats AMR poses to human and animal health and environmental resilience, there is *prima facie* reason to believe we ought to employ policies to manage AMR. However, there are a range of policies available to choose from. We hold to a pluralist approach (Dawson, 2016), claiming that which policy is ethically preferable will depend on a number of different factors, such as how restrictive it is, how effective it is, whether it is proportionate, whether it distributes burdens in an acceptable way etc. What’s more, these values may trade off against each other, for instance a policy that is more effective at managing AMR may plausibly also be more restrictive than alternatives, as creating the most change in AMR-associated behaviours often both imposes more burdens to achieve behaviour change, and is more effective due to that greater (if successful) behaviour change. To determine which policy for managing AMR is ethically preferable, there is both more ethical work needed to support particular arguments, and, importantly, more empirical work needed to provide evidence for the empirical premises within these arguments.

In the ethics literature, the duties that states bear toward their citizens to curb AMR have been discussed in depth, as have tensions between individual freedoms and collective goals (Malmqvist and Munthe, 2020). Yet, there has been little consideration of how ethical arguments for favouring particular policies often rest on empirical premises, and what this implies for the directions that empirical work ought to take. To discuss the duties that states and other entities bear toward their citizens in the context of managing AMR, we might refer to moral concepts like collective moral responsibility, where a state might have a duty to impose penalties for failures by responsible parties, and effectiveness, where a state might appropriately seek the most effective policy to better prevent the harms of AMR befalling its citizens. We argue that establishing *effectiveness* relies on evidence of bad AMR-related outcomes and links to practices that cause them, as well as alternatives that would avoid them. We also argue that establishing *moral responsibility* relies on evidence of causal links between certain practices and AMR-related outcomes, evidence of the availability of knowledge concerning how common practices contribute to AMR, and evidence of responsible agents’ capacities to undertake alternative practices. Without sufficient evidence, these empirical premises in the arguments from moral responsibility and from effectiveness cannot together contribute to justifying a state’s preferring certain policies to enforce (AMR-associated) duties over others. Recently, more work has been done to address this issue, including considering AMR as a set of ‘super-wicked’ problems—i.e. problems that are urgent, whose proposed solvers are weak institutional bodies and contributors to the problem. These problems require a coordinated response (Littman *et al.*, 2020). But the super-wicked problems approach has not been used much by policymakers as a tool to ethically evaluate or choose between policies. A more fruitful approach might be for policymakers to consider ethical justifications for preferring more restrictive policies to manage AMR (insofar as liberty is not the only value of importance, in keeping with a pluralist approach (Dawson, 2016)). They might then call for the empirical research to be done that is needed to support an ethical preference for one policy over another.

In the literature as it stands, there is still not consensus on how to defend an ethical preference for one AMR-addressing policy over another. Some policies fall under a single reservoir approach, where a policy involves an intervention in only one context in which AMR may emerge. Other policies which we may compare these to fall under a ‘One Health’ approach (Wieland and Robichaud, 2017). The One Health approach to AMR might be considered a management framework that acknowledges and engages with the role of cross-reservoir transmission in AMR spread, although several, sometimes conflicting, definitions of a One Health approach exist (Ryu *et al.*, 2017; Bhatia, 2019; Mackenzie and Jeggo, 2019). The policies that align with this management framework, those that are designed for managing AMR across reservoirs and using tools from multiple policy areas, might be termed ‘One Health policies’ and may include multiple interventions. Most definitions of One Health emphasise an interdisciplinary framework to combat AMR, including with respect to policy formulation and implementation. That is, under the One Health approach, policies are informed by research in multiple disciplines, and may occur at local, national and global policy levels, and in policy areas including the environment, public health, agriculture and others. Whilst there has been early ethical analysis of One Health, including some discussion of whether a One Health approach demands a moral framework of its own (Johnson and Degeling, 2019), the connections between this work and empirical work have not been thoroughly explored.

The expansiveness of the policy areas and levels that a One Health approach includes means that particular policies that fall under the One Health umbrella may be more restrictive than many single-reservoir or single-policy area alternatives. We might therefore have some reason to reject them, or at least not prefer them. But this might not be ethically justified if there are more or more weighty *pro tanto* moral reasons to prefer the more restrictive or coercive policy.

To illustrate, within the UK and some EU countries, there is surveillance of both healthy and diseased livestock (Varona *et al.*, 2020). Practically, these policies may include ‘improvements in antimicrobial use regulation and policy, surveillance, stewardship, infection control, sanitation, animal husbandry and alternatives to antimicrobials’ (McEwen and Collignon, 2018: 2) They may target single or multiple reservoirs, provided they recognise the threat of cross-reservoir transmission. Regulation and stewardship may restrict the actions of health care professionals, veterinarians, farmers and consumers, which may count against the policy, ethically. Yet, it may be more effective to ensure antimicrobials are used carefully across all these areas, thus reducing harm to the population from bad AMR-associated outcomes. There are many other factors to consider, too—the implementation of One Health policies is further complicated by conflicts of interest between policy areas or between different (health) threats in the same policy area, by moral dilemmas that arise before or during implementation (Johnson, 2021), and by our current limited scientific understanding of AMR transmission. Whilst ethical analysis based on these considerations may be somewhat lacking, One Health policies are already being implemented. Further ethical analysis is needed to catch up with this development.

Consider how policymakers might be guided toward an overall solution by considering points for and against a particular policy, if they consider multiple moral factors—for instance, perhaps the precautionary principle and the principle of the least restrictive alternative. Considering the precautionary principle, we might identify a reason to prefer One Health policies over alternatives, because a cross-reservoir approach seems intuitively like it would be more effective and more proportionate to the threat of serious and imminent harm from AMR (even lacking scientific certainty surrounding the harm) (Steel, 2014). Indeed, arguments have already been made for various interventions such as registration of veterinary uses of Fluoroquinolones and other antibiotics in some countries on the basis of the precautionary principle (Cheng, 2012). However, conflicting with the judgement that might be made on the basis of considering the precautionary principle alone, we might also have a reason to prefer single-reservoir policies that are less restrictive, if we also consider the moral concept of the least restrictive alternative (Byskov, 2019), as many One Health policies are more restrictive (Ibrahim *et al.*, 2019). We do not intend to make claims about how the precautionary principle and the principle of the least restrictive alternative might weigh against each other here. Instead, we accept an initial presumption in favour of the least restrictive alternative among the policies available, and as such, that the burden of justification for ethically preferring a more restrictive policy lies squarely with that more restrictive policy and the other moral concepts that might, together, justify preferring it instead (in this case, that more restrictive alternative is likely a One Health policy).

Whilst the precautionary principle is one consideration that may weigh in favour of One Health policies, others exist that have not been as well-explored. We wish to help remedy the lack of adequate justification for burdensome policy alternatives with this paper. First, we hold that preferring a more restrictive alternative (like a One Health policy) over another may be ethically justified if there are weightier moral reasons^2^ to undertake it than the alternative. Whilst there may be many *pro tanto* moral reasons for preferring a One Health policy over alternatives, two key considerations are effectiveness and moral responsibility. We focus on these two throughout this paper, as they particularly highlight the need for better connections between empirical work and philosophical work in order to justify policy choices surrounding AMR. We will assume that, all else equal, the balance can be tipped in favour of preferring a more restrictive (One Health) policy over a less restrictive alternative if the more restrictive policy is significantly more effective at achieving the public health goal, and imposes burdens in a way that more accurately tracks collective moral responsibility for relevant public health outcomes. As we will argue in this paper, policymakers might, then, prefer a more restrictive One Health policy to a less restrictive single-reservoir alternative. We will say, for short-hand, that these two ethical considerations can together provide an ethical justification for the preference. Our second point is that a closer relationship between scientific and ethical work is needed to evidence these claims. Further development on the basis of our two points in this paper may help in the evaluation of existing policies in specific instances when a more restrictive alternative has been preferred to a less restrictive one for managing AMR, or vice versa. This is an essential future goal, given that, as ethicists Jasper Littman and Adrian Viens claim, many ‘guidelines on antibiotic stewardship focus on technical considerations [...] but neglect to consider the moral questions underpinning and guiding what a good steward of antimicrobials should do’ (Littmann and Viens, 2015: 210). As such, it is important that we establish an ethical rationale for choosing such guidelines and policies, both considering the ethical arguments, and the empirical work on which they rely.

Certain directions of scientific research need further development, to evidence arguments based on effectiveness and moral responsibility that may ethically justify preferring One Health policies to address AMR over less restrictive alternatives. We focus on a livestock farming case study here, as it is one of the largest areas of antimicrobial use outside human medicine (Manyi-Loh *et al.*, 2018; Ibrahim *et al.*, 2019), and is closely related to human health and environmental resilience. Similar discussion could be useful for other reservoirs and policy instances.

In this paper, we first offer general discussion of AMR, One Health and farming practices that may contribute to AMR, in the first section. We go on to present an overall ethical justification for One Health policies over alternative, often less restrictive policies. This justification might feature two key arguments, one from effectiveness and one from moral responsibility. Each argument relies on empirical and ethical premises. The first argument, concerning effectiveness, is then discussed. The second argument, concerning moral responsibility (both accountability for past AMR-associated outcomes and responsibility for future AMR management) is discussed next. We conclude that when defensible empirical and ethical premises feature in these arguments, these effectiveness and responsibility arguments might contribute to an ethical justification for implementing One Health policies over alternatives. This ethical justification may be strong enough to outweigh considerations of the least restrictive alternative and overcome a preference for less restrictive single-reservoir policies. Of course, our ethical justification may not apply to cases where a One Health policy is less restrictive than alternatives. One Health policies may sometimes place very few burdens or restrictions on action. And conversely, single-reservoir policies may sometimes impose more intrusive restrictions, perhaps on more people. In such cases, the ethical justification may be less necessary, as there is less of a presumption against One Health policies to begin with if they are the least restrictive among the alternatives. However, arguments from effectiveness and responsibility may still strengthen the justification for undertaking the One Health policy further, by providing additional *pro tanto* moral reasons for pursuing it.

## Livestock Farming and Its Impact on the Emergence of AMR

Implementation of One Health policies is particularly challenging in the context of livestock farming. Globally, within these reservoirs, human-critical classes of antimicrobials are routinely used as therapies, prophylactics, and in some cases to promote livestock growth. (Although, there was an EU/UK-wide ban of livestock growth-promoting antibiotics from 1 January 2006 (Koluman and Dikici, 2013).) The increasing prevalence of livestock AMR poses a threat to animal health, as well as raising potential concerns regarding food-supply sustainability, and the livelihoods of commercial farmers (Acar and Moulin, 2013; Paul and Varghese, 2020). Furthermore, putative AMR-associated transmission events from livestock to other reservoirs pose potential harm to humans and other animals, and may negatively impact environmental resilience. Both occupational contact (e.g. farmers, vets), and the consumption of food products can lead to cross-reservoir AMR transmission, e.g. in the case of livestock-associated methicillin-resistant *Staphylococcus aureus* (LA-MRSA) (Anjum *et al.*, 2019). Each of these harms ought to be accounted for in ethical justifications for more restrictive policies to manage AMR in the livestock farming case. Considering these harms aligns with the increasing acceptance of more types of morally relevant harms, exhibited particularly in the literature on animal welfare and on climate change ethics. This is exhibited, e.g. in the UK Nuffield Council on Bioethics’ recent report on genome editing in livestock, which took an approach that relied heavily on considerations of the environment and animal and human welfare, and how these might be impacted by genome editing livestock animals (Nuffield Council on Bioethics, 2021). To complicate matters further, livestock farming practices are globally diverse, and vary in the desired product, consumer demands, geography, labour availability and technology, as well as cultural and legal factors. Different practices include intensive, extensive, organic and nomadic farming, all of which pose unique challenges for justifying unified One Health policies across geographic contexts. Put together, the limited availability of (context-specific) empirical evidence and the diversity of livestock reservoirs highlights a significant knowledge gap. Filling this gap is important not only for scientific advancement, but for the ethical justification for One Health policies.

An ethical justification of One Health policies over less restrictive alternatives might rely on a number of arguments, but the particular potential justification we have in mind relies on two arguments. First, that One Health policies are more effective than alternatives at addressing bad outcomes of AMR (thus preventing more human and animal suffering, and environmental destruction). Second, that the imposition of burdens under One Health policies better that tracks the attribution of two kinds of moral responsibility for AMR than alternatives:

(1) Accountability for past bad outcomes (e.g. increasing the risk of AMR transmission events or the amount of AMR) by those (in this context, farmers, consumers or the state) who undertake certain practices. We claim that it is better to impose burdens or restrictions on groups that contribute to those outcomes via, e.g. One Health policies.
(2) Forward-looking responsibility for AMR management strategies (e.g. reducing risk of future cross-reservoir transmission events) by those who have the capacity to act.^3^ We claim that more burdens regarding preventing future AMR-associated bad outcomes should be imposed on potential contributors to those strategies via, e.g. One Health policies.

**Figure 1.**
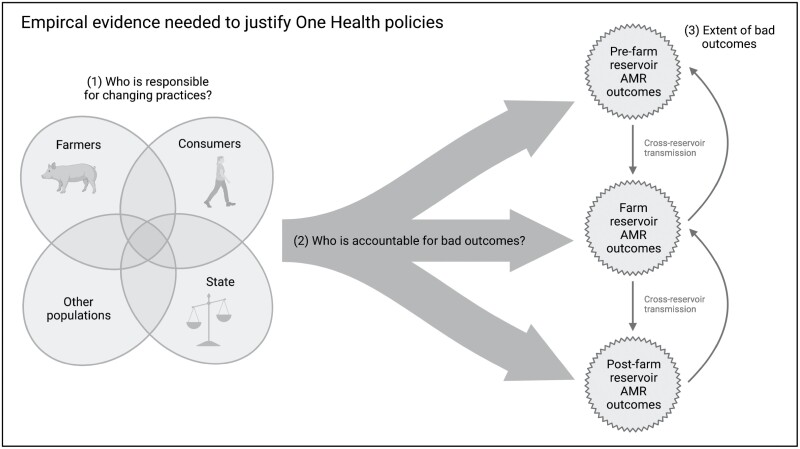
A diagrammatic representation of the empirical evidence needed to justify One Health policies. Populations have influence over practices and one another (1), who then cause AMR outcomes (2), which can be measured empirically (3)

A greater volume and quality of empirical evidence at local, national and global levels may better support an ethical justification for One Health policies over less restrictive alternatives. Each argument involves empirical premises. That is, to ethically justify (or evaluate) One Health policies, we require empirical evidence, which can be divided into three categories—namely, evidence underpinning: (i) who (e.g. farmers, consumers, the state) is able to change existing practices to avoid future bad outcomes; (ii) who is causally responsible for past bad outcomes and to what extent, including influence of farming practices/state legislation/consumers on types/rates of AMR evolution and transmission; and (iii) the extent of bad outcomes, including quantifying the risk of transmission events, characterising rates/types of AMR evolution, patient morbidity/mortality rates and healthcare/other economic costs. These categories of evidence are represented in Figure 1.

## Justifying One Health

To help illustrate our ethical justification for preferring more restrictive One Health policies over less restrictive alternatives in the sections that follow, consider a comparison between two hypothetical policies for managing the spread of the *X*, a highly mobile gene which confers extended-spectrum cephalosporin resistance, which is known to have originated from intensive farming. First, we have Policy 1, a single-reservoir policy employed in healthcare. It examines the effects of increasing nosocomial outbreaks of *X*-positive bacterial strains in a country, resulting in excess human mortality. Policymakers acknowledge this bad outcome within this single human reservoir, and trace responsibility for the deaths only within the human healthcare system, back to factors such as antibiotic overuse, poor sanitation and poor infection control. To combat the problem, Policy 1 is implemented, restricting the use of extended-spectrum cephalosporin antibiotics to treat *X*-positive bacterial infections to only those most in need. Policy 2 is a One Health policy. It, too, examines the effects of *X*-positive bacterial strains in terms of increasing human mortality from nosocomial outbreaks in the country. However, it also acknowledges the existence of livestock with similar resistant strains to the hospital outbreaks. It traces the problem back to the transmission of the resistant strains from livestock to humans (exacerbated by antimicrobial use in the healthcare system). This cross-reservoir transmission is made possible by farming practices wherein contaminated food is undetected and then consumed. In response, Policy 2 is implemented (successfully, and in line with the recognition of more relevant reservoirs). It constitutes a bundle of policies across areas. The first, Policy 2.1, addresses the use of antimicrobials in animals to begin with by prohibiting extended-spectrum cephalosporin use in livestock farming. Second, Policy 2.2 imposes fines for continued extended-spectrum cephalosporin use in livestock farming. Third, Policy 2.3 imposes a tax on farmers’ use of other non-last resort antimicrobials that might increase extended-spectrum cephalosporin resistance through co-selection, and therefore still require stewardship. Finally, Policy 2.4, similarly to Policy 1, addresses doctors’ prescription behaviours by capping their extended-spectrum cephalosporin prescription numbers. Policy 2 also acknowledges that since gene *X* has already been selected for, it is unlikely to disappear completely. By intervening in multiple reservoirs, Policy 2 seeks to at least stabilise, if not reduce, gene *X* levels.

At this stage, note that the One Health policy bundle is clearly more restrictive, and maybe harder to justify than the single-reservoir policy, Policy 1, if we accept that the burden of justification lies with a more restrictive policy. It traces (possibly) more tenuous links between agriculture and healthcare and imposes coercive measures in both public health and agriculture systems. Yet, it may also be *more effective*, in acknowledging and addressing the causes of bad outcomes across policy areas and across reservoirs (insofar as the policy is implemented successfully in line with the recognition of more cross-reservoir transmission). It may therefore prevent more harms, providing a moral reason for preferring this more effective policy. Policy 2 also seems to impose burdens in a morally preferable way that tracks moral responsibility for bad AMR-associated outcomes. It does not merely impose restrictions on doctors (as Policy 1 does). This may be morally preferable because doctors are not the only ones who are either morally responsible for having caused or contributed to causing existing bad outcomes, nor the only ones who are able to impact future outcomes and who might be held responsible for doing so in future. Penalising the imposition of harms seems appropriate both as a matter of justice, and as a deterrent, each aspects of responsibility-tracking as a moral reason for preferring Policy 2. Policy 2 considers the role of farmers and consumers of factory-farmed meat in causing bad outcomes and imposes burdens to ensure that they will take responsibility for past outcomes, and take responsibility to prevent them in future.^4^

## The Argument from Effectiveness

In the overall ethical justification for preferring One Health policies like Policy 2 over single-reservoir alternatives like Policy 1, the first identifiable argument is that from effectiveness, containing the following premises (P) and conclusion (C):

**P1:** There are bad outcomes spread across reservoirs from increasing AMR
**P2**: Single-reservoir policies neglect to recognise or address cross-reservoir transmission
**P3**: Successful One Health policies recognise and address cross-reservoir transmission
**P4:** A policy that recognises and addresses more bad outcomes is more effective than alternatives
**C:** Successful One Health policies are more effective than single-reservoir policies

Let us begin with the first premise. P1 claims that there are bad outcomes from increasing AMR, occurring across reservoirs. Consider, then, empirical data necessary to defend P1 in this particular case. For the gene *X* farm outbreak, empirical data largely falls into two main categories: bad outcomes within the farm (e.g. reduced animal health, farmer illness, mobile genetic contexts supporting the dissemination of gene *X*, and economic costs), and the bad outcomes of transmission events from the farm livestock to other reservoirs (e.g. *X*-positive bacteria in contaminated meat products, contaminated surface run-off or contaminated slurry used as fertiliser). We might also note that, from hypothetical global *X* spread, mortality is increased from both extended-spectrum cephalosporin-resistant infections, and other deaths from a redirection of healthcare resources towards these infections. It is this empirical data that is needed to defend P1, and thus support the argument from effectiveness.

This empirical premise provides a grounding to which we can add descriptive claims P2 and P3, that single-reservoir policies do not recognise or aim to address cross-reservoir outcomes and that One Health policies do. We assume the truth of these premises on the basis of the definitions of One Health compared to single-reservoir policies as presented in the section above. Building on this, P4 claims that a policy that is successfully implemented and that adequately recognises and addresses more bad outcomes, across more reservoirs is more effective than alternatives. By acknowledging bad outcomes across reservoirs and tracking the causes of bad outcomes across reservoirs, we identify more routes by which AMR can be combated, and thus may *more effectively* address bad outcomes than when only one of multiple contributing factors and reservoir-specific outcomes are acknowledged and addressed.^5^ Recognising bad outcomes leads to more effective policy only under certain conditions, including a state’s ability and motivation to enact and enforce burdensome AMR-associated policies, and the connection between recognising and addressing bad outcomes from AMR. The mere recognition without action is not sufficient to make for more effective policy. Presumably, however, policymakers consider implementing AMR management policies from the motivation of wishing to address AMR, so as long as other considerations (e.g. economic limits) allow, recognising more bad outcomes likely means more bad outcomes are addressed via policies like the One Health bundle Policy 2, compared to those addressed via single-reservoir policies like Policy 1.

This might be an ethical premise, not merely a descriptive one. That claim relies on a link between effectiveness and harm reduction. Insofar as harm reduction is a morally desirable thing, then that premise and the conclusion of the argument are both ethical: policymakers have moral reason to choose a more effective policy.

Let us flesh out that link further. The goal of managing or reducing AMR is a moral goal, in part precisely because of the bad outcomes of AMR. Harms, including, say, the loss of human life, increased pain and suffering from disease, and reduced social contact and mental health (perhaps due to measures to curb disease spread, like lockdowns) are all potentially morally bad under mainstream moral theories. Harms are likely reduced, then, when policy more effectively manages AMR.^6^ The policy that is most effective is ethically preferable in these consequentialist terms.^7^ (We go on to consider a non-consequentialist moral reason with our discussion of responsibility-tracking, below.) What’s more, to justify the link between effectiveness and ethical preference, we can refer to the issue of resource allocation in resource-limited systems (Daniels, 2016), wherein difficult decisions must be made about devoting resources to supporting different public health campaigns/policies based, in part, upon how effective they are expected to be. Managing AMR via antimicrobial stewardship in farming, healthcare and other settings appears to be an area where there is substantial harm that could be reduced, with measures that are cost-effective (World Bank, 2017).

If these premises hold and if our link between effectiveness and moral reasons is accepted, then effectiveness is one moral reason in favour of One Health policies being ethically preferable to alternative policies.^8^

We turn now to a second contributing moral reason underlying an overall justification for a One Health policy over the alternatives: that it is preferable because it better tracks moral responsibility for bad outcomes.

## The Argument from Responsibility

Let’s return to our example of a country considering whether to implement a One Health policy or a single-reservoir alternative for managing the spread of gene *X*. We are establishing an ethical argument for implementing a bundle of One Health policies like Policy 2—focussed on addressing behaviours that contribute to *X* prevalence across reservoirs—over Policy 1—focussed on addressing extended-spectrum cephalosporin resistance outcomes in healthcare, via healthcare policies alone. In this section, we explore the ethical justification for preferring One Health policies like Policy 2 over less restrictive single-reservoir alternatives like Policy 1 based on the argument from responsibility. This argument concerns the appropriate attribution of two types of moral responsibility: backward-looking (or accountability-type) moral responsibility, and forward-looking (or task-type) moral responsibility (Smiley, 2017). The ethical justification for preferring a more restrictive policy is stronger if that policy imposes burdens in line with the accurate and appropriate recognition of moral responsibility for past and future (bad) AMR outcomes. Below, we outline and defend the responsibility-tracking argument.

First, however, some background on responsibility. The responsibility we discuss here is *moral* in that it includes a normative element—we are not merely discussing, say, causal responsibility. What’s more, when it comes to AMR, it is the aggregation of many uses of antimicrobials that contribute to their overuse and subsequent ineffectiveness. That means that the responsibilities for bad AMR-associated outcomes are *collective*—not merely individual—to align with the multiple contributions likely necessary to cause bad outcomes. No single person is attributed responsibility for a (past or future) outcome (Giubilini, 2019: ch. 2), because their actions *alone* have not caused it.^9^

Consider backward-looking responsibility, or accountability, first. As footnoted above, we consider the imposition of burdens via policy bundles like Policy 2 to be appropriate when they are placed on agents who can be held accountable for having contributed to AMR-associated harms. This linking of burdens and accountability follows a combination of David Shoemaker’s discussion of accountability as connected to disregard for others (Shoemaker, 2015), Matthew Talbert’s explanation of capacity requirements (Talbert, 2019), and Larry May’s work on group intentionality (May, 1987). For accountability according to a combination of these conceptions, on top of causality we must establish (i) the agent’s capacity to have avoided causing a given (bad) outcome; (ii) their capacity to understand the bad outcomes of their action; and (iii) their having collectively and intentionally undertaken the action anyway, showing disregard for others’ interests. Where these requirements are satisfied, we might impose burdens, according to Shoemaker’s discussion of appropriate responses to accountability. Indeed, Shoemaker claims that ‘to be accountable for something is to be liable to being appropriately held to account for it, which is to be eligible for a range of fitting responsibility responses with a built-in confrontational element’. (Shoemaker, 2015: 88) Whilst Shoemaker only explores accountability at the individual level, Deborah Tollefsen takes this further, in discussing the reactive attitudes we hold and burdens we impose on collective agents that we hold accountable (Tollefsen, 2003). Appropriate burdens in reaction to accountability for bad outcomes might come in the form of policies that restrict actions or impose financial costs on the responsible populations.

Note that all the requirements must be fulfilled for the imposition of responsibility-tracking burdens to be morally appropriate. For example, a group of children may not be held accountable for bad outcomes they have caused, assuming the group lacks the capacity to fully understand the consequences of their actions. If the group of children was aware in advance of how their classroom disruption affected others’ learning, and had the capacity to act differently as a group, yet were intentionally and collectively disruptive anyway, then they might appropriately have burdens imposed on them as an accountable group (May, 1987). In the AMR context, a One Health policy bundle might impose burdens on a group^10^—say, farmers—for past contributions to AMR for which they are collectively causally responsible via their farming practices, for which they can be held accountable because of the existence of alternative farming practices that do not contribute to AMR, because of their having had knowledge that their farming practices may contribute to AMR, and because of their having gone ahead with the harmful farming practices anyway. Policy 2 would hold farmers accountable in this case via Policy 2.2, imposing fines for continued extended-spectrum cephalosporin use in livestock farming. Such a policy can be further defended by reference to the protection of future generations, as Littman and Viens claim:

‘If the preservation of effective antimicrobials is in the interest of current and future generations, and indeed their lives depend on it, then we should also hold people blameworthy or sanctionable for the ignorant, unnecessary, or wrongful use of antibiotics, or any other practice that is likely to hasten the emergence of AMR. This may mean that we will be morally justified in imposing greater burdens or costs for their contribution to the current state of AMR’. (Littman and Viens, 2015: 211)

The argument from responsibility also concerns the imposition of burdens via a One Health policy in accordance with the attribution of forward-looking responsibility. Forward-looking responsibility differs a little from accountability: it concerns not past, but future outcomes. In the AMR context, forward-looking responsibility might be attributed to groups that have the capacity to enact change in outcomes. Policy that imposes burdens in line with the attribution of forward-looking responsibility might involve taxation or restrictions on use, like the taxes imposed on farmers for antibiotic use in Policy 2.3. Attributing forward-looking responsibility requires that the agent(s) can be expected to cause certain outcomes via their actions, that they are aware of these outcomes, and that they have the capacities to make changes that cause good outcomes (say, AMR management) or avoid bad outcomes (say, contributions to cross-reservoir transmission events). In addition to the burdens aligning with the attribution of forward-looking responsibility that Policy 2.3 makes, Policy 2.4 imposes burdens on doctors by capping their last-resort antibiotic prescription numbers. Policy 1 does the same, but it does not hold farmers responsible in a forward-looking sense for their potential future contributions to AMR-associated outcomes for humans from extended-spectrum cephalosporin use in livestock farming and subsequent transmission events.

Having discussed these two types of collective moral responsibility, we turn now to defending the premises necessary to attribute backward-looking and forward-looking collective moral responsibility, and support the argument from responsibility-tracking.

We might outline the argument as follows:

**P1:** There are bad outcomes spread across reservoirs from increasing AMR
**P2:** Collective agents have caused these bad AMR-associated outcomes
**P3:** Those agents could avoid causing these bad outcomes via alternative practices
**P4:** Those agents could plausibly know about the potential bad outcomes and alternative practices
**P5:** Single-reservoir policies neglect to burden agents that (may) cause bad AMR-associated outcomes where the cause is in another reservoir
**P6:** One Health policies burden agents that (may) cause bad AMR-associated outcomes, even where the cause is in another reservoir
**P7:** A policy that burdens agents who (may) cause bad AMR-associated outcomes is more accurately responsibility-tracking in how it imposes burdens compared to alternatives
**C:** One Health policies impose burdens that more accurately track collective moral responsibility compared to single-reservoir policies

First, consider P1. This is an empirical claim shared by the argument from effectiveness. It relies on empirical work to support it, as discussed above. P2 adds an element of causation, and will also require empirical work, as well as philosophical assumptions regarding connections between causation and agency (Talbert, 2019). P3 and P4 are (partially empirical) requirements for attributing moral responsibility, and we return to these below. P5 and P6 contrast where One Health policies and single-reservoir policies impose burdens, wherein, by the definition of each term, there are multiple groups upon whom burdens are imposed for One Health policies, and only one group for single-reservoir policies. P7 does much of the explanatory work. It is a descriptive claim that holds when it is the same individual or group that is responsible and has burdens imposed on them. Finally, in support of the ethical argument, a link is needed between accurate responsibility-tracking and moral reason to implement a policy. The discussion of accountability-type responsibility we engaged in at the start of this section explains why it is morally better to attribute moral responsibility in proportion to the causation of previous bad outcomes, as with the case of farmers who have causally contributed to bad AMR-associated outcomes by unnecessarily feeding their livestock antibiotics when alternatives like less intensive farming are available. Policies that impose burdens that more accurately track accountability to group agents across reservoirs might be considered ethically preferable because institutions like the state are expected to penalise wrong actions, and reward right actions. Where actions that have been taken that harm others by contributing to bad AMR-associated outcomes, states may, therefore, do better to penalise these actions through policy, insofar as the other requirements for establishing moral responsibility for those harms are fulfilled.

Let us return to P3 and P4. For both premises, the groups that take actions that affect AMR (either in the past or in the future) must be identified. The premises also rely on data that shows what portion of a bad outcome is due to the actions of these groups. They allow for the attribution of both backward-looking and forward-looking responsibility. Causation is the primary empirical element required for accountability, and feasible alternative actions are the primary empirical element required for forward-looking responsibility. What kinds of empirical evidence can establish, first, causal links between collective agents and past bad outcomes? What kinds can establish, second, the existence of feasible alternative actions that could be taken to avoid future bad outcomes?

First, consider the identification of the relevant populations as collective agents. Whilst this may include farmers in our case study, we want to also acknowledge here the power of consumers and the state to impact farming practices that can contribute to transmission events. After all, according to theories of epistemic responsibility, ‘[a]s information about the private benefits and social costs of using antibiotics in farm animals becomes more widely available, consumers have an increasing responsibility to act on it by changing their purchasing habits and trying to persuade governments to make it harder to purchase meat from animals unnecessarily dosed with antibiotics’. (Anomaly, 2020: 303) If evidence shows that educational resources are available, publicised and reasonably accessible, consumers may have a responsibility to become aware of the disparities in antimicrobial usage in farming methods, such as intensive farming which led to gene *X* dissemination, compared to farming practices which limit antimicrobial use. Despite the higher cost of non-intensively farmed meat products, consumers may have a responsibility to either spend more, or reduce meat consumption.

Building on the identification of relevant populations (e.g. the state, farmers, consumers), studies to support the accountability and forward-looking responsibility arguments for One Health policies would need to characterise the outward transmission rate/type of resistances, and destination reservoirs. These characterisations are likely to vary for different AMR mechanisms, in different settings over time. However, the rate and type of AMR transmission *into* farms must also be studied, to avoid confounded conclusions and accurately assess farmers accountability. For the gene *X* example, empirical data may include farming practices for therapies/prophylactics which can be shown to select for third-generation cephalosporin resistance, livestock feed regulations implemented by farmers and regulators that contribute to disease spread, slurry usage regulations, nearby water treatment regulations, policies on screening of meat for contamination and demand for meat. This accurate attribution of causal responsibility, along with ethically relevant elements such as capability to act or regulate, goes on to support the claim that it is responsibility-tracking to impose burdens in alignment with the attribution of moral responsibility. The claim that there is also moral reason to do so is one we have defended above. Thus, holding farmers, consumers and others responsible using policies like those in the Policy 2 bundle is more responsibility-tracking, and there is moral reason to opt for this policy, compared to alternatives, insofar as the other premises and our link between responsibility and ethical preference hold.

## Conclusion

In this paper, we have considered the ethical justification for preferring One Health policies to less restrictive single-reservoir policy alternatives. The justification consists of two key arguments, the first based on effectiveness, and the second based on burdens tracking responsibility. Both rest partly on empirical premises, and thus are limited by our current directions for scientific research and the extent to which they provide the necessary evidence. We argue that at least some scientific work should be guided by the need to supply evidence for these ethical arguments. If there is already at least some reason to expect One Health policies to be preferable to single-reservoir policies, then this must be backed up by whatever context-specific data that can be produced that considers bad AMR-related outcomes across reservoirs, and the causation of these outcomes across reservoirs.

To develop the overall ethical justification for preferring a One Health policy bundle to a less restrictive single-reservoir alternative, we explored the argument from effectiveness. We showed how the argument relies on empirical work including, e.g. studying *X*-positive bacteria in contaminated meat products. Furthermore, we explored the argument from responsibility, and how this rests on, first, empirical work to establish causal links between past bad outcomes and cross-reservoir AMR-related practices, and second, empirical work to establish the existence of alternative practices that could be implemented to avoid contributing to AMR in the future. Empirical evidence for this argument could include, e.g. identifying farming practices, therapies and prophylactics which can be shown to select for colistin resistance, and the existence of viable alternatives that could be employed instead. When empirical and ethical premises are all defensible, the effectiveness and responsibility arguments have the potential to, together, form a strong ethical justification for preferring One Health policies over single-reservoir alternatives. Whilst empirical work alone, or even a single argument from effectiveness or responsibility alone (containing both empirical and ethical premises) is insufficient to ethically justify preferring One Health policies over less restrictive alternatives, both arguments put together, relying on both empirical work and philosophical work may give policymakers reason to morally prefer a One Health policy to a less restrictive alternative like a single-reservoir policy.

Whilst we have discussed how other moral reasons might count for or against preferring One Health policies, one issue we have not considered is how the two arguments we explore should be weighed, where they conflict. For example, a One Health policy might be more effective than a single-reservoir alternative, but also less accurately responsibility-tracking (and therefore, perhaps, morally worse), if it seems to mis-attribute responsibility. In such cases, how should we proceed? Answering the question would be another paper in itself. Indeed, there are even further ethical questions left to be addressed:^11^ Who is responsible for implementing One Health policies and managing AMR? How should One Health policies operate under limited resources? How does a One Health policy to address AMR intersect with other moral environmental concerns like climate change? How can we pool funding more efficiently under One Health policies rather than traditional, policy-sector-specific funding? One Health policies may generate cost savings compared to single-reservoir alternatives, due to shared tools and outcomes across policy areas (World Bank, 2017). These further questions are beyond our remit here, but this work starts us on a path to addressing them once ethical justifications for One Health policies over less restrictive alternatives is fully established.

## Footnotes

1 This paper will only discuss bacterial AMR, but more generally, the term also refers to other AMR such as viral, fungal etc.

2 The moral reasons we consider here are evaluative concepts (as opposed to deontic concepts). We assume, then, that the right can be defined in terms of the good, and that which policies a state should implement depends on what produces the most good for residents of that state (and perhaps outsiders, too).

3 In this context, this would include farmers with access to and ability to implement alternative practices, consumers driving market demand for meat products and states that can impose policies to ensure those practices are taken up.

4 This imposition of burdens in alignment with the attribution of responsibility for agents who increase risks to others aligns with previous arguments concerning the responsibility-tracking imposition of burdens and payments for compensation in situations of social risk imposition, particularly relating to health. We assume, on this basis, that imposing burdens on agents in proportion to their moral responsibility (as well as according to the most effective reduction of bad outcomes) makes a policy morally preferable to alternatives that are, on this definition, less accurately responsibility-tracking, and perhaps, therefore, morally worse.

5 Note that the prevention or reduction of some of the bad AMR-associated outcomes identified as part of this process may be reframed as promotion of good AMR-associated outcomes, insofar as minimising the harms of resistant infections can be considered a benefit. However, in the policy area of antimicrobial stewardship in high-income countries, there are unlikely to be benefits aside from harm minimisation, unlike in other AMR areas such as research and development, where the development of new treatments, e.g. phage therapies, might be considered as a standalone benefit.

6 That is, all else being equal, i.e. there not being any overriding opportunity costs or financial costs, rights violations or other moral concerns that make addressing AMR, overall, a worse option than not addressing it.

7 Some argue that a consequentialist lens is appropriate, particularly in the public health sphere, where harms and benefits to large populations are clear and relevant to ethical analysis (Giubilini *et al.*, 2018; Anomaly, 2020), whist others take the view that harms ought not be used in ethical analysis when the persons being harmed are mere statistical persons, and are not identifiable, as in the public health sphere. To this, we would respond that ‘statistical lives matter as much as identifiable lives’. (Savulescu *et al.*, 2020: 626). This applies equally to public health emergencies like pandemics, and urgent but longer-term concerns like AMR.

8 Indeed, effectiveness has already been acknowledged in previous work as a key aspect of justifying certain AMR management interventions (Nijsingh *et al.*, 2020).

9 That is, in most cases, where resistance does not emerge in a particular host who goes on to spread it and cause significant bad outcomes only through their own overuse of antimicrobials and spreading behaviours, a scenario which seems very unlikely. The same attribution of collective responsibility for collectively-caused outcomes applies in other cases, such as vaccination.

10 Note that here, we refer to a group that is more directly responsible for bad AMR-associated outcomes. There may be highly diffuse or indirect causes, for which it is more difficult to identify an agent or track causation. In these cases, there may be empirical difficulties, but our point remains that insofar as an agent can be held accountable, this provides an ethical justification for burdensome policies that track this moral responsibility.

11 For more on these, see Ribeiro *et al.* (2019).
